# Risk factors and prognostic factors for pulmonary large cell neuroendocrine carcinoma with brain metastasis

**DOI:** 10.1002/cam4.5267

**Published:** 2022-09-20

**Authors:** Xiaoyun Chen, Yedong Huang, Fangrong Chen, Hui She, Xiangqi Chen

**Affiliations:** ^1^ Department of Respiratory and Critical Care Medicine Fuzhou Second Hospital Fuzhou People's Republic of China; ^2^ The Third Clinical Medical College Fujian Medical University Fuzhou People's Republic of China; ^3^ Department of Gynecology Oncology Fujian Maternity and Child Health Hospital, Affiliated Hospital of Fujian Medical University Fuzhou People's Republic of China; ^4^ Department of Respiratory Medicine Fujian Key Laboratory of Translational Research in Cancer and Neurodegenerative Diseases (Fujian Medical University Union Hospital) Fuzhou People's Republic of China

**Keywords:** brain metastasis, large cell neuroendocrine carcinoma, nomogram, prognostic factors, risk factors

## Abstract

**Background:**

As the studies regarding the brain metastasis (BM) of pulmonary large cell neuroendocrine carcinoma (LCNEC) are insufficient, the present research aims to describe the risk factors and prognostic factors that are related to cancer‐specific survival (CSS) for LCNEC patients with BM.

**Methods:**

The data of LCNEC patients between January 2010 and October 2018 were obtained from the SEER database. Binary logistic regression analyses were utilized to screen the possible risk factors related to BM. Prognostic factors for LCNEC patients with BM were indentified by Cox regression analyses. Moreover, a nomogram was established to predict the 6‐, 12‐, and 18‐month CSS rates. The concordance index (C‐index), receiver operating characteristic (ROC) curves and calibration curves were utilized to assess the discrimination and reliability of the model. Clinical decision curves (DCAs) were used to evaluate the clinical benefits and utility of our model.

**Results:**

Totally, 1875 patients were enrolled, with 294 (15.7%) of them having BM at diagnosis. Multivariate logistic regression analyses revealed that patients with age < 65 (odds ratio, OR = 1.564) and N2 staging (OR = 1.775) had a greater chance of developing BM. Age (≥ 65 vs. < 65: hazard ratio, HR = 1.409), T staging (T1 vs. T0: HR = 4.580; T2 vs. T0: HR = 6.008; T3 vs. T0: HR = 7.065; T4 vs. T0: HR = 6.821), N staging (N2 vs. N0: HR = 1.592; N3 vs. N0: HR = 1.654), liver metastasis (HR = 1.410), primary site surgery (HR = 0.581) and chemotherapy (HR = 0.452) were independent prognostic factors for LCNEC patients with BM. A nomogram prediction model was constructed by incorporating these factors. Using the C‐index, calibration curves, ROC curves, and DCAs, we found that the clinical prediction model performed well.

**Conclusion:**

We described the risk factors and prognostic factors that were associated with CSS for LCNEC patients with BM. The related nomogram was established and validated to help clinicians formulate more rational and effective treatment strategies.

## Graphical abstract

This is the first large population study for LCNEC patients with BM. The related prognostic model based on these factors can guide clinical treatment decisions and prognostic judgments.

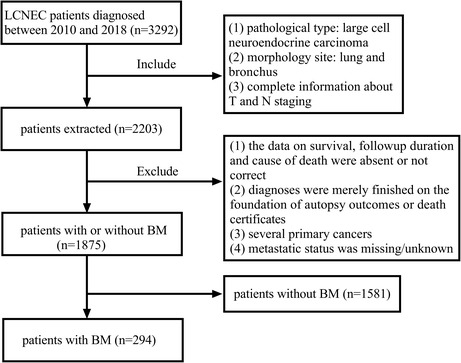

## 1 INTRODUCTION

Pulmonary large cell neuroendocrine carcinoma (LCNEC) is a rare type of non‐small cell lung cancer (NSCLC), accounting for 1–3% of lung cancer.^1^, ^2^, ^3^ LCNEC was reclassified as a subgroup of pulmonary neuroendocrine tumors in 2015,^4^ which included small cell lung cancer (SCLC), typical carcinoid and atypical carcinoid. LCNEC shows robust malignant behavior and invasive ability.^3^, ^5^, ^6^ About 50% of patients with advanced LCNEC will develop brain metastasis (BM).^7^, ^8^ Many patients develop neurological symptoms, which usually lead to serious damage to cognition and quality of life.^9^, ^10^, ^11^ Generally, the survival time of such patients is merely a few months.^12^ The optimal treatment for symptomatic BM are mainly based on local approaches, including surgical resection, stereotactic radiosurgery (SRS) and whole brain radiotherapy (WBRT) which can prevent or slow down the growth of BM.^13^ The high morbidity and harmfulness of BM make it imperative to explore related risk factors and prognostic factors of BM in LCNEC patients in order that physicians can early identify these patients and offer adequate treatment without delay.

The nomogram model is an intuitive scoring system formed by integrating different variables, which can optimize the prediction accuracy of individuals.^14^ At present, the model has been widely used in the prognostic evaluation of various diseases, but no research has been completed to construct a prediction model for BM in LCNEC patients. Therefore, data from the Surveillance, Epidemiology, and End Results (SEER) data base were used to explore the risk factors and prognostic factors that were related to cancer‐specific survival (CSS) for LCNEC patients with BM. Moreover, a nomogram was established to guide clinical treatment decisions and prognostic judgments.

## 2 MATERIALS AND METHODS

### 2.1 Population selection

**FIGURE 1:**
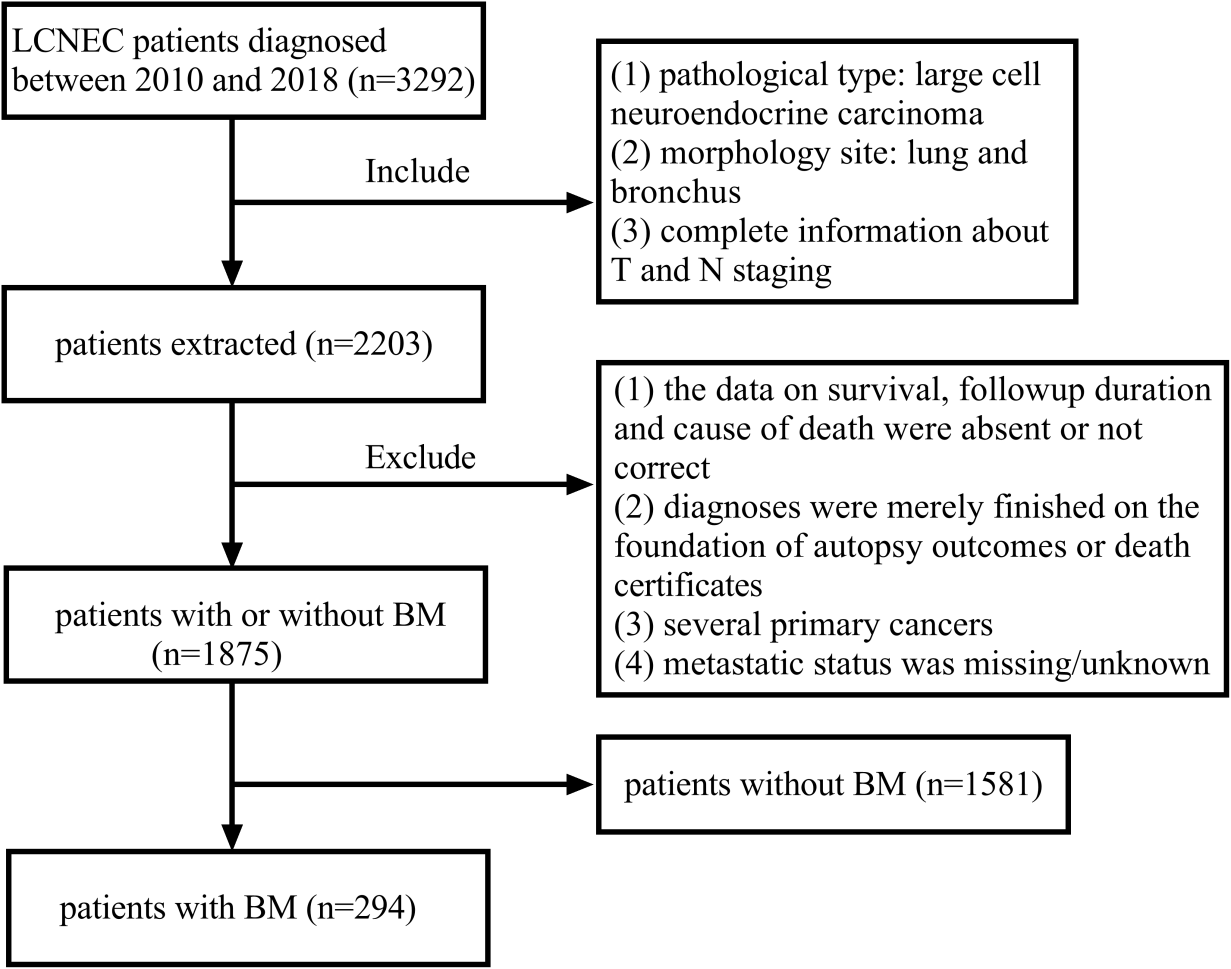
The flow chart of selection

Patient information was acquired from the SEER database. A total of 1875 LCNEC patients between January 2010 and October 2018 were obtained. The inclusion criteria were: (1) pathological type: large cell neuroendocrine carcinoma (ICD‐O‐3 histologic type: 8013/3); (2) morphology site: lung and bronchus; and (3) complete information about T and N staging. In contrast, the exclusion criteria were (1) the data on survival, follow‐up duration and cause of death were absent or not correct; (2) diagnoses were merely finished on the foundation of autopsy outcomes or death certificates; (3) several primary cancers; (4) metastatic status was missing/unknown. According to the presence or absence of BM at diagnosis, we divided the cases with no metastasis of brain into the non‐BM group (n = 1581) and those with brain metastasis into the BM group (n = 294). The flow chart of selection is illustrated in Figure 1.

### 2.2 Data collection

Four variables were extracted according to demographic characteristics, including age at diagnosis(< 65, ≥ 65 years), gender (males or females), race (white, black, and others), and marital status at diagnosis (married or unmarried). Eight variables were extracted according to cancer characteristics, including primary sites (main bronchus, upper lobe, middle lobe, lower lobe, and others), laterality (left, right, and unknown), T staging (T0, T1, T2, T3, and T4), N staging (N0, N1, N2 and N3), bone metastases (no or yes), liver metastases (no or yes), lung metastases (no or yes) and AJCC seventh edition staging (I, II, III, IV and unknown). Three variables were extracted according to treatment regimens, including primary site surgery (no/unknown or yes), radiotheraphy (no/unknown or yes) and chemotherapy (no/unknown or yes). Cancer specific survival (CSS) is considered to be the time from diagnosis to death due to LCNEC. In this article, CSS was utilized as the main endpoint.

### 2.3 Statistical analyses

The basic clinicopathological data of each group were analyzed by the chi‐square test to show the differences between the non‐BM and BM groups, which were displayed as frequencies and proportions (%). Univariate logistic regression analyses were utilized to screen the possible risk factors related to BM. The variates with *p* < 0.05 in the univariate logistic regression analyses were included in multivariate logistic regression analyses. Survival differences were evaluated by the Kaplan–Meier method and Log‐rank test. The prognostic factors with significant differences related to CSS in univariate Cox regression analyses were verified by multivariate Cox regression analyses. The nomogram was then built by the ‘rms’ package in the R software based on independent prognostic factors. The discrimination degree of the nomogram was assessed via the concordance index (C‐index) and receiver operating characteristic (ROC) curves. At the same time, the calibration degree of the nomogram was tested by drawing calibration curves to ensure the accuracy and reliability. Additionally, clinical decision curves (DCAs) were used to evaluate the clinical benefits and utility of our model. This study used the SPSS Statistics software 25.0 to perform the chi‐square test, univariate logistic regression analysis, multivariate logistic regression analysis, survival analysis, univariate Cox regression analysis and multivariate Cox regression analysis. Calibration curves, ROC curves and DCAs were drawn using the R language software 4.1.3. *p* < 0.05 was considered statistically significant.

## 3 RESULTS

### 3.1 Baseline features

Totally, 1875 patients were enrolled, and 294 (15.7%) of them were in the BM group while 1581 (84.3%) of them were in the non‐BM group. The BM group was significantly younger (51.7% vs. 39.5%) than the non‐BM group (*p* < 0.001). Statistical analysis revealed that remarkable diversities existed in T and N staging between BM and non‐BM patients (*p* < 0.001). What's more, the BM group had more bone metastasis (22.8% vs. 12.8%), liver metastasis (23.5% vs. 14.7%) and lung metastasis (17.0% vs. 10.4%) compared with non‐BM group (*p* < 0.001). In terms of treatment, BM patients had less primary site surgery (7.8% vs. 43.1%, *p* < 0.001) and more radiotherapy (78.2% vs. 32.7%, *p* < 0.001). Additionally, the distribution of gender, race, marriage, primary site, laterality and chemotherapy was not associated with statistically significant outcomes (*p* > 0.05). The baseline features are presented in Table 1.

**TABLE 1:** Baseline characteristics of LCNEC patients divided by BM (*n* = 1875)

Variable	BM, *n* (%) *n* = 294 (15.7)	No BM, *n* (%) *n* = 1581 (84.3)	Total, *n* (%) *n* = 1875	*p*‐value
Age (years)				<0.001
< 65	152 (51.7)	624 (39.5)	776 (41.4)	
≥ 65	142(48.3)	957 (60.5)	1099 (58.6)	
Gender				0.334
Female	144 (49.0)	726 (45.9)	870 (43.2)	
Male	150 (51.0)	855(54.1)	1005 (53.6)	
Race				0.535
White	252 (85.7)	1314 (83.1)	1566 (83.5)	
Black	32 (10.9)	207 (13.1)	239 (12.7)	
Others	10 (3.4)	60 (3.8)	70 (3.7)	
Marriage				0.487
Married	156 (53.1)	804 (50.9)	960 (51.2)	
Unmarried	138 (46.9)	777 (49.1)	915 (48.8)	
Primary Site				0.198
Main bronchus	14 (4.8)	65 (4.1)	79 (4.2)	
Upper lobe	156 (53.1)	912 (57.7)	1068 (57.0)	
Middle lobe	12 (4.1)	63 (4.0)	75 (4.0)	
Lower lobe	74 (25.2)	404 (25.6)	478 (25.5)	
Others	38 (12.9)	137 (8.7)	175 (9.3)	
Laterality				0.059
Left	112 (38.1)	642 (40.6)	754 (40.2)	
Right	170 (57.8)	909 (57.5)	1079 (57.5)	
Unknown	12 (4.1)	30 (1.9)	42 (2.2)	
T stage				<0.001
T0	5 (1.7)	13 (0.8)	18 (1.0)	
T1	46 (15.6)	444 (28.1)	490 (26.1)	
T2	82 (27.9)	487 (30.8)	569 (30.3)	
T3	80 (27.2)	325 (20.6)	405 (21.6)	
T4	81 (27.6)	312 (19.7)	393 (21.0)	
N stage				<0.001
N0	90 (30.6)	726 (45.9)	816 (43.5)	
N1	32 (10.9)	151 (9.6)	183 (9.8)	
N2	127 (43.2)	493 (31.2)	620 (33.1)	
N3	45 (15.3)	211 (13.3)	256 (13.7)	
Bone Metastasis				<0.001
No	227 (77.2)	1378 (87.2)	1605 (85.6)	
Yes	67 (22.8)	203 (12.8)	270 (14.4)	
Liver Metastasis				<0.001
No	225 (76.5)	1349 (85.3)	1574 (83.9)	
Yes	69 (23.5)	232 (14.7)	301 (16.1)	
Lung Metastasis				0.001
No	244 (83.0)	1416 (89.6)	1660 (88.5)	
Yes	50 (17.0)	165 (10.4)	215 (11.5)	
Stage				‐
Unknown	0 (0.0)	96 (6.1)	96 (5.1)	
I	0 (0.0)	329 (20.8)	329 (17.5)	
II	0 (0.0)	146 (9.2)	146 (7.8)	
III	0 (0.0)	239 (15.1)	239 (12.7)	
IV	294 (100.0)	771 (48.8)	1065 (56.8)	
Primary site surgery				<0.001
No/Unknown	271 (92.2)	899 (56.9)	1170 (62.4)	
Yes	23 (7.8)	682 (43.1)	705 (37.6)	
Radiotherapy				<0.001
No/Unknown	64 (21.8)	1064 (67.3)	1128 (60.2)	
Yes	230 (78.2)	517 (32.7)	747 (39.8)	
Chemotherapy				0.071
No/Unknown	126 (42.9)	768 (48.6)	894 (47.7)	
Yes	168 (57.1)	813 (51.4)	981 (52.3)	

Abbreviation: BM, brain metastasis.

Risk factors for developing BM.

The clinical characteristics of age, gender, race, marriage, primary site, T staging and N staging were studied via univariable logistic regression analyses. Then the risk factors that might be related to BM were involved in multivariable logistic regression analyses. Eventually, the outcomes revealed that patients with younger age (OR = 1.564, 95% CI: 1.215–2.013) and N2 staging (OR = 1.775, 95% CI: 1.306–2.412) had a higher incidence of BM (*p* < 0.05), while those with T1 staging (OR = 0.329, 95% CI: 0.110–0.980) had a lower incidence of BM (*p* > 0.05). The details are displayed in Table 2.

**TABLE 2:** Risk factors associated with BM in LCNEC patients (*n* = 1875)

Variable	Univariate logistic		Multivariate logistic	
	OR (95% CI)	*p*‐value	OR (95% CI)	*p*‐value
Age (years)				
≥ 65	Reference		Reference	
< 65	1.642 (1.279–2.108)	<0.001	1.564 (1.215–2.013)	0.001
Gender				
Female	Reference			
Male	0.885 (0.689–1.135)	0.334		
Race				
White	Reference			
Black	0.806 (0.543–1.198)	0.286		
Others	0.869 (0.439–1.720)	0.687		
Marriage				
Married	Reference			
Unmarried	0.915 (0.713–1.175)	0.487		
Primary site				
Main bronchus	Reference			
Upper lobe	0.794 (0.435–1.450)	0.453		
Middle lobe	0.884 (0.380–2.059)	0.776		
Lower lobe	0.850 (0.454–1.594)	0.613		
Others	1.288 (0.652–2.542)	0.466		
T stage				
T0	Reference		Reference	
T1	0.269 (0.092–0.789)	0.017	0.329 (0.110–0.980)	0.046
T2	0.438 (0.152–1.261)	0.126	0.498 (0.171–1.452)	0.202
T3	0.640 (0.222–1.847)	0.409	0.703 (0.241–2.050)	0.518
T4	0.675 (0.234–1.948)	0.467	0.676 (0.232–1.971)	0.474
N stage				
N0	Reference		Reference	
N1	1.709 (1.101–2.654)	0.017	1.546 (0.992–2.411)	0.054
N2	2.078 (1.550–2.786)	<0.001	1.775 (1.306–2.412)	<0.001
N3	1.720 (1.166–2.539)	0.006	1.308 (0.867–1.972)	0.200

Abbreviations: CI, confidence interval; OR, odds ratio.

### 3.2 Survival outcomes and prognostic factors

**FIGURE 2:**
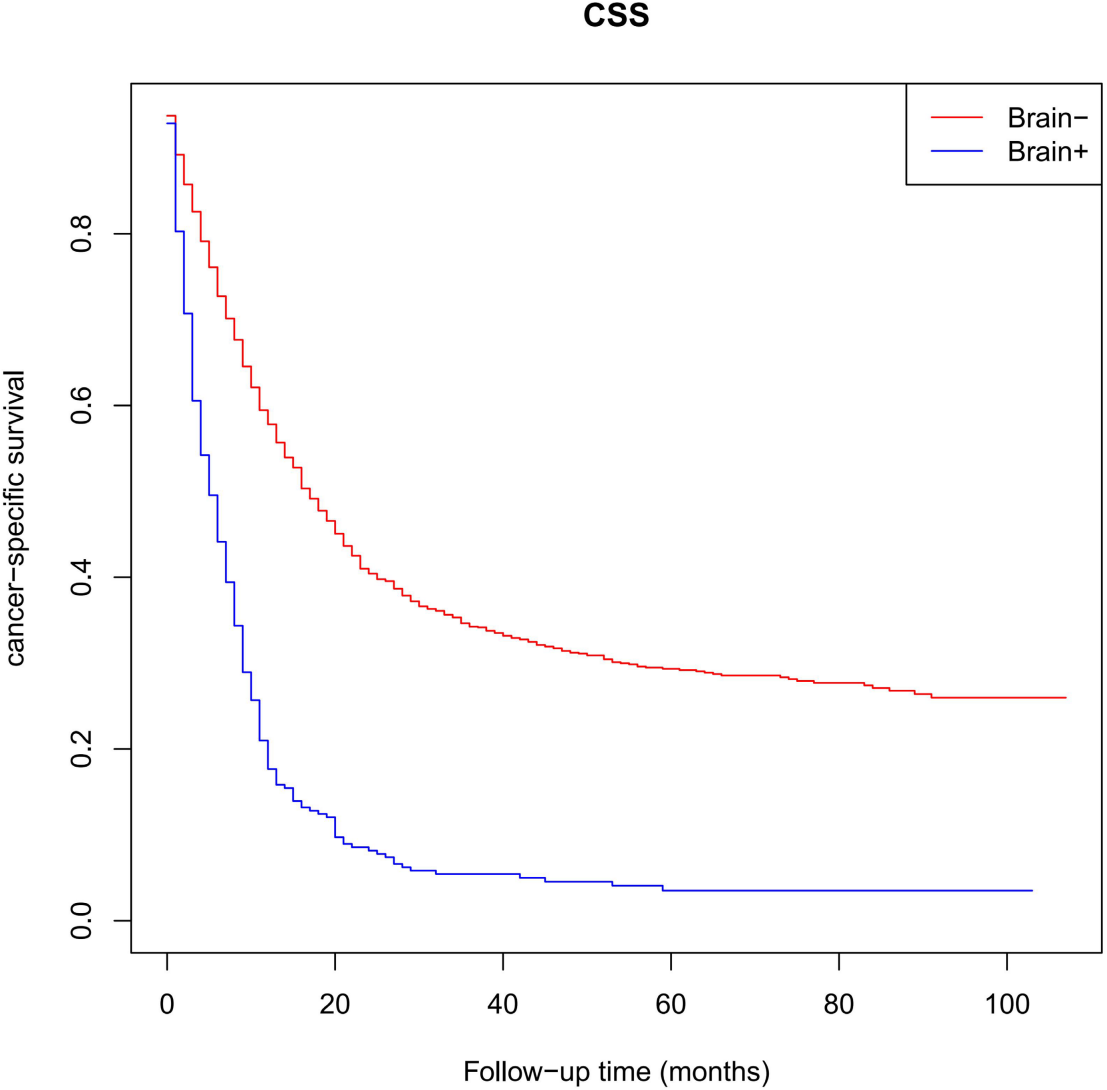
Kaplan–Meier curves of CSS for LCNEC patients with or without BM

As presented in Figure 2, survival analyses revealed that BM patients had significantly lower CSS compared with non‐BM patients (5 months vs. 17 months, *p* < 0.001). Therefore, LCNEC patients with BM often exhibited unfavorable outcomes. The concrete outcomes of the median survival are presented in Table 3. In univariable Cox regression analyses, age, T staging, N staging, bone metastasis, liver metastasis, primary site surgery, radiotheraphy and chemotherapy were the predictors of CSS for LCNEC patients with BM (*p* < 0.05). Subsequently, multivariable Cox regression analyses indicated that an increasing age (hazard ratio, HR = 1.409), T staging > T0 (T1 vs. T0: HR = 4.580; T2 vs. T0: HR = 6.008; T3 vs. T0: HR = 7.065; T4 vs. T0: HR = 6.821), N staging > N1 (N2 vs. N0: HR = 1.592; N3 vs. N0: HR = 1.654) and liver metastases (HR = 1.410) were independent poor prognostic factors (HR >1, *p* < 0.05). On the other hand, primary site surgery (HR = 0.581) and chemotherapy (HR = 0.452) were independent favorable prognostic factors (HR <1, *p* < 0.05). The complete outcomes are presented in Table 4.

**TABLE 3:** Survival data for LCNEC patients divided by BM (*n* = 1875)

	Patients, *N* Median CSS 95% CI, Months	
No BM	1581	17 (15.314–18.686)
BM	294	5 (3.944–6.056)
*p*‐value		<0.001

Abbreviations: BM, brain metastasis; CI, confidence interval; CSS, cancer‐specific survival.

**TABLE 4:** Univariate and multivariate Cox analyses of CSS in the eligible patients. (*n* = 294)

Characteristics	Univariate		Multivariate	
	HR (95% CI)	*p*‐value	HR (95% CI)	*p*‐value
Age (years)				
< 65	Reference		Reference	
≥ 65	1.483 (1.162–1.894)	0.002	1.409 (1.092–1.819)	0.008
Gender				
Female	Reference			
Male	1.102 (0.868–1.400)	0.424		
Race				
White	Reference			
Black	0.785 (0.536–1.150)	0.214		
Others	0.766 (0.406–1.445)	0.410		
Marriage				
Married	Reference			
Unmarried	1.171 (0.922–1.488)	0.196		
Primary site				
Main bronchus	Reference			
Upper lobe	1.117 (0.644–1.936)	0.694		
Middle lobe	0.939 (0.425–2.073)	0.876		
Lower lobe	1.372 (0.771–2.441)	0.282		
Others	1.059 (0.563–1.991)	0.859		
T stage				
T0	Reference		Reference	
T1	6.017 (1.449–24.976)	0.013	4.580 (1.093–19.197)	0.037
T2	6.247 (1.527–25.566)	0.011	6.008 (1.460–24.719)	0.013
T3	7.369 (1.802–30.145)	0.005	7.065 (1.717–29.072)	0.007
T4	8.506 (2.078–34.821)	0.003	6.821 (1.655–28.118)	0.008
N stage				
N0	Reference		Reference	
N1	1.429 (0.941–2.172)	0.094	1.388 (0.902–2.136)	0.136
N2	1.622 (1.214–2.167)	0.001	1.592 (1.168–2.170)	0.003
N3	1.683 (1.159–2.443)	0.006	1.654 (1.114–2.455)	0.013
Bone metastasis				
No	Reference		Reference	
Yes	1.727 (1.298–2.299)	<0.001	1.190 (0.865–1.638)	0.285
Liver metastasis				
No	Reference		Reference	
Yes	1.490 (1.124–1.937)	0.005	1.410 (1.026–1.937)	0.034
Lung metastasis				
No	Reference			
Yes	1.314 (0.958–1.802)	0.090		
Primary site surgery				
No/Unknown	Reference		Reference	
Yes	0.510 (0.323–0.807)	0.004	0.581 (0.360–0.937)	0.026
Radiotherapy				
No/Unknown	Reference		Reference	
Yes	0.617 (0.462–0.823)	0.001	0.779 (0.580–1.046)	0.097
Chemotherapy				
No/Unknown	Reference		Reference	
Yes	0.514 (0.403–0.657)	<0.001	0.452 (0.346–0.591)	<0.001

Abbreviations: CI, confidence interval; HR, hazard ratio.

### 3.3 Prognostic nomogram construction and validation

**FIGURE 3:**
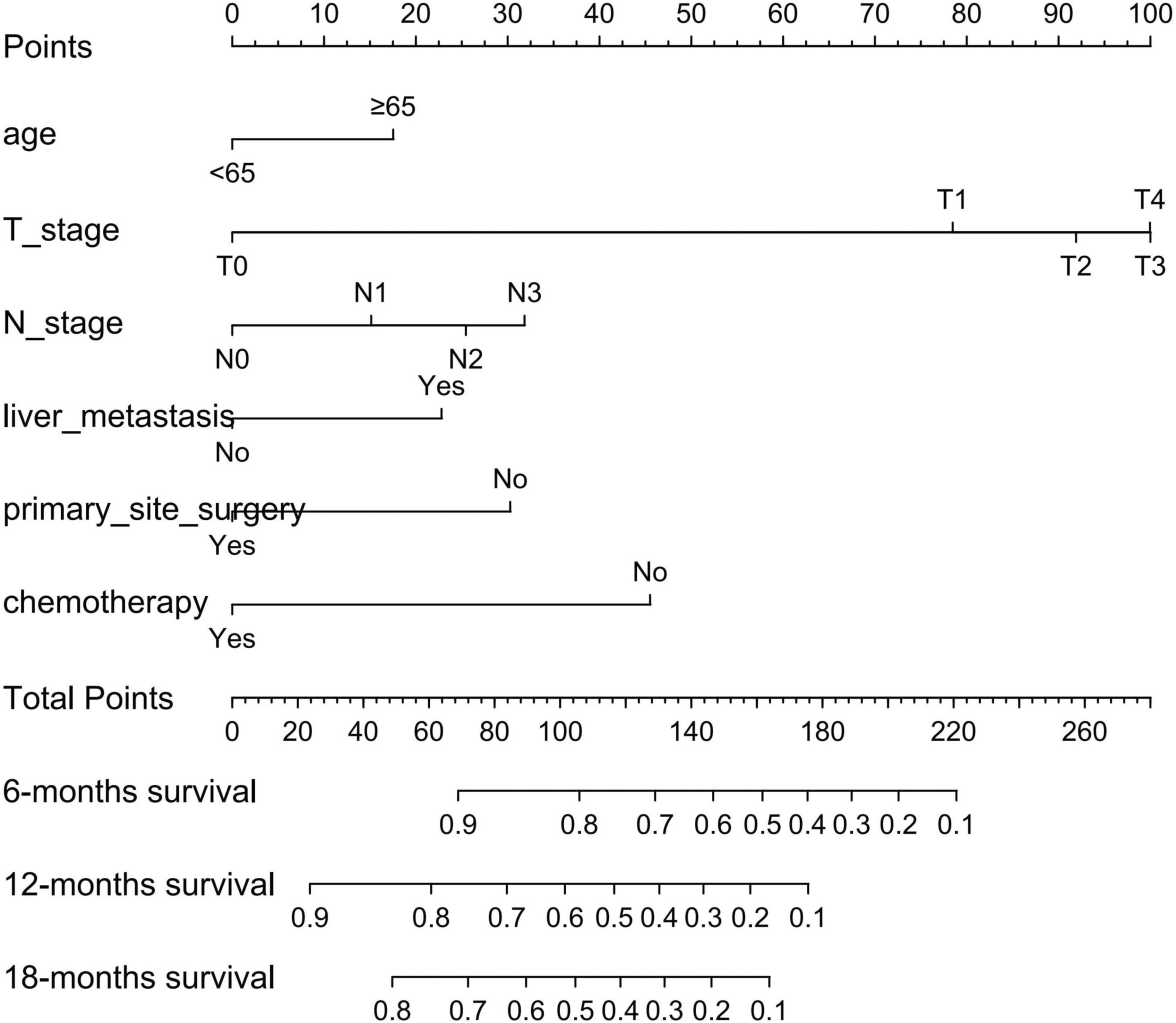
Nomogram for predicting the 6‐month, 12‐month, and 18‐month CSS rates of LCNEC patients with BM

**FIGURE 4:**
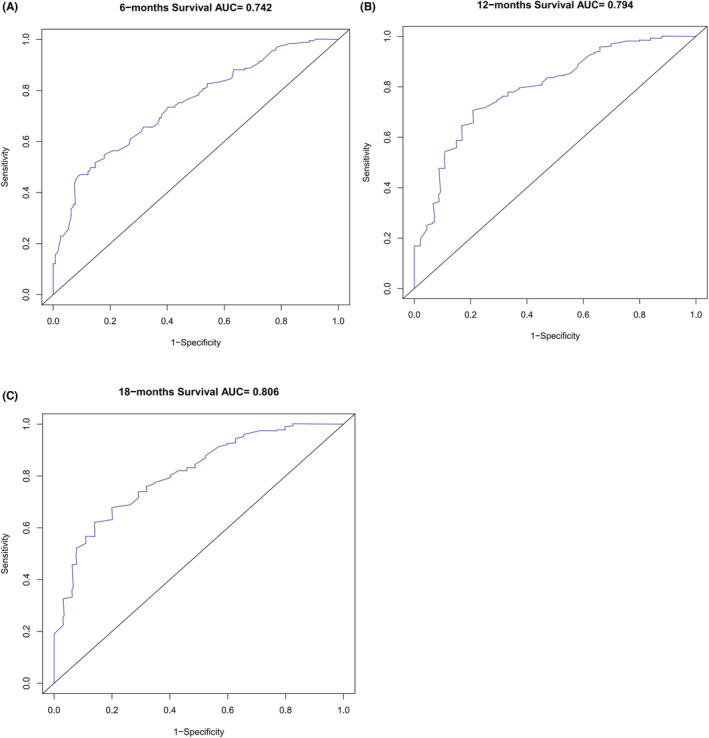
Receiver operating characteristic curves (A, B, C)

**FIGURE 5:**
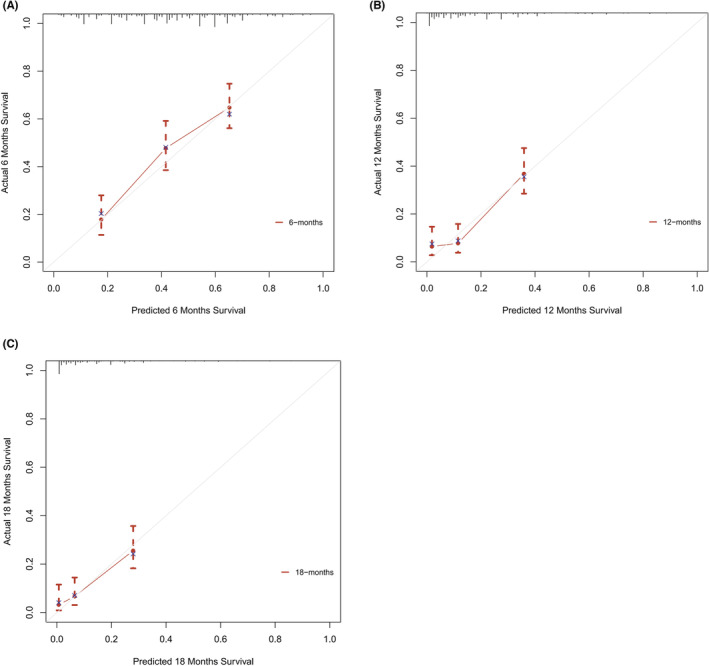
Calibration plots for predicting the 6‐month, 12‐month, and 18‐month CSS rates of LCNEC patients with BM (A, B, C)

**FIGURE 6:**
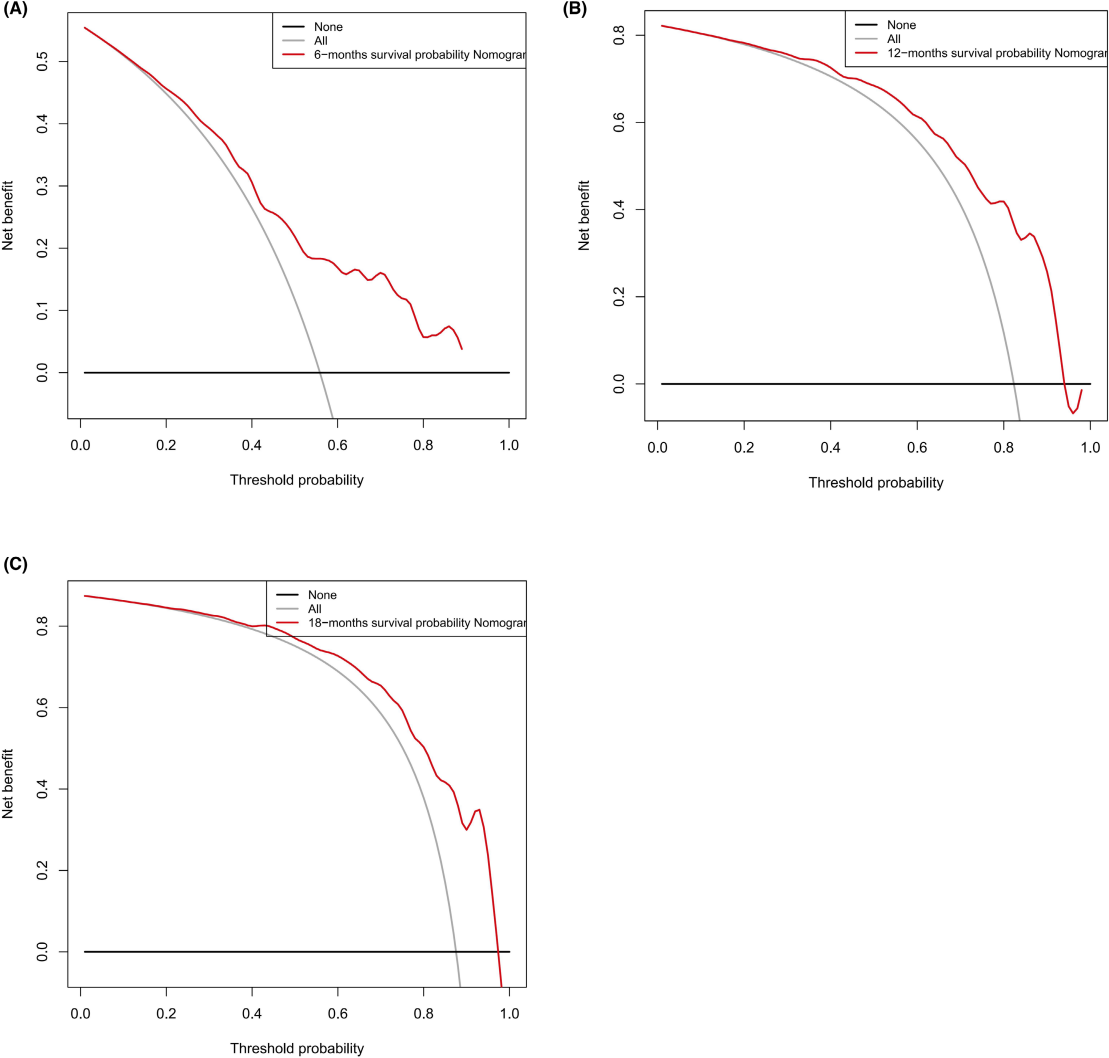
Decision curve analyses of the nomogram (A, B, C)

Based on the 6 aforementioned prognostic factors, we established a CSS nomogram to forecast the survival probability of LCNEC patients with BM, as shown in Figure 3. The scoring for every variable was added together to obtain an overall score to predicted the 6‐, 12‐, and 18‐month survival probability for each patient. It was interesting that T staging had the greatest impact on prognoses, followed by N staging and chemotherapy. The C‐index of the nomogram was 0.712 (95% CI: 0.677–0.747). The area under ROC curves (AUC) for predicting the 6‐month, 12‐month, and 18‐month CSS rates was 0.727, 0.776 and 0.78 (Figure 4), respectively, showing good predictive accuracy. At the same time, the calibration curves for the 6‐, 12‐, and 18‐month CSS rates were all close to the ideal 45° reference line (Figure 5), suggesting a high consistency between the predicted and actual values. DCA analyses revealed that the predictive model displayed satisfactory clinical benefits and utility (Figure 6).

## 4 DISCUSSION

LCNEC is a highly aggressive malignancy prone to brain metastasis.^7^, ^8^ Currently, this is the first large population study for LCNEC patients with BM. Our findings showed that 15.7% of LCNEC patients developed BM. Younger age and N2 staging were associated with a greater chance of developing BM. In addition, age, T staging, N staging, liver metastasis, primary site surgery and chemotherapy were independent prognostic factors for LCNEC patients with BM. The related prognostic model based on these factors can guide clinical treatment decisions and prognostic judgments.

Substantial literatures indicated that younger NSCLC patients were more prone to brain metastasis.^15^, ^16^ Consistent with these reports, our findings showed that patients with younger age had a higher incidence of BM. It is considered that younger patients often live longer, hence they have more time to develop BM. Besides, as lymph nodes are usually the first stop for cancer cells to metastasize and cancer cells can also spread through the lymphatics into the brain,^17^ we discovered that LCNEC patients with BM tended to have more N2 staging than non‐BM patients. Thereby, inhibiting lymphatic spread may be an effective method to prevent distant metastasis.^18^ Experiments have shown that lymphatic vessels can provide a diffusion pathway in mouse models, but whether this form of cancer cell spread exists in humans needs to be verified.^19^, ^20^

In terms of prognostic factors in LCNEC patients with BM, several previous studies suggested that age > 65 years was related to poor prognosis.^21^, ^22^, ^23^ Our study also observed significant differences regarding the prognosis between older patients and younger patients. Most elderly patients have underlying diseases, such as hypertension, diabetes and other chronic diseases. Some of them are bedridden for a long time, which often results in poor physical status. In the traditional TNM staging system, lung cancer patients will survive longer with earlier T and N staging, which is supported by our prediction model. Our study identified T staging > T0 as an independent poor prognostic factor. The increase in T staging means an increase in tumor volume, which is a manifestation of further malignant tumor progression. What's more, we discovered that patients with N2 and N3 staging had lower survival rates than patients with N0 staging. We speculate that lymphatics may be the way for tumor cells to spread, which aggravates the survival of patients. Consistent with the finding of Liu CF et al,^13^ our study also found that liver metastases in LCNEC patients with BM shortened the survival time. The occurrence of liver metastases often indicates that the tumor is more aggressive, hence the prognosis is relatively poorer.

Nowadays, the standard therapeutic regimen for LCNEC patients with BM is still uncertain.^8^, ^24^, ^25^ Our study hold that primary site surgery treatment is an important component of the comprehensive treatment in advanced pulmonary LCNEC, which is consistent with the results of several previous studies.^26^, ^27^, ^28^ If primary site surgery is not possible, chemotherapy can be considered. The pathological features of LCNEC are similar to SCLC, whereas it is classified as NSCLC. Physicians can choose the NSCLC regimen and SCLC regimen. Several studies have shown that the SCLC regimen is more effective. Four to six cycles of etoposide in combination with either cisplatin or carboplatin are usually recommended for advanced LCNEC patients.^29^, ^30^, ^31^ However, the research of Nicole Roberts et al showed no statistically significant difference between the two chemotherapy regimens.^32^ During the last decade, targeted therapy for oncogene‐dependent diseases and immunotherapy for patients with PD‐L1 positive cancers have transformed the treatment options for NSCLC. For pulmonary LCNEC, target gene mutations are predominantly found in mixed forms of LCNEC‐adenocarcinoma, while ‘pure’ LCNEC target gene mutations are rare. It has been reported that LCNEC patients with EGFR mutations may benefit from gefitinib treatment.^33^ For patients with ALK rearrangements detected in LCNEC, the efficacy of TKI therapy is conflicting.^34^, ^35^ Although PI3K/AKT/mTOR pathway alterations are frequently detected in LCNEC,^36^, ^37^ unfortunately, no effective targeted drugs have been developed. Delta‐like protein 3 is an inhibitory Notch ligand expressed in approximately 65% of LCNEC, which provides an excellent target for the development of antibody–drug conjugates (ADCs). Compared with SCLC and low‐grade neuroendocrine tumors, LCNEC has a higher level of PD‐L1 expression.^38^, ^39^ In a case report of Chauhan et al, 3 LCNEC patients with unsatisfactory platinum‐based chemotherapy results were treated with nivolumab immunotherapy and achieved objective response or stable disease.^40^ An advanced LCNEC patient with a high tumor mutation burden level was reported in another case, who achieved a complete response to nivolumab treatment.^41^ These studies provided insights into the efficacy of immunotherapy. To date, targeted therapy or immunotherapy are not indicated for patients with LCNEC. Clinical trials involving LCNEC are strongly recommended in order to determine an optimal treatment strategy and correlate biomolecular properties with the promising involvement of new therapeutic options.

As a visual tool for cancer prognostic assessment, the nomogram has been used to evaluate the prognosis of LCNEC patients and demonstrates good predictive value. However, the model that can forecast the prognosis of LCNEC patients with BM has not been developed yet. In this study, age, T staging, N staging, liver metastases, primary site surgery and chemotherapy were incorporated into the prediction model according to the outcomes of multivariable Cox regression analyses, and a nomogram was established. The C‐index and AUC were high and the calibration curves for the 6‐, 12‐, and 18‐month CSS rates showed good consistency, which revealed that the model had satisfactory discrimination and calibration. Therefore, it has certain value for clinicians to judge the prognosis of LCNEC patients with BM.

However, there are some deficiencies. First, as our study utilized retrospective data from the SEER database, the results require the substantiation from more prospective trials. Second, the data on smoking status, comorbidities, performance scores, targeted therapies and immunotherapies were absent due to the constraints of the SEER database, which might affect survival results. Third, although the predictive model established in this study showed good accuracy and clinical applicability, patients were not divided into modeling groups and validation groups for model verification because the number of cases in some subgroups was too small. Eventually, as we did not have sufficient data from other sources due to the rarity of BM, external validation is required to realize further corroboration in the future.

## 5 CONCLUSION

Our study described the risk factors and prognostic factors that were associated with CSS for LCNEC patients with BM. The related nomogram was established and validated to help clinicians formulate more rational and effective treatment strategies.

## AUTHOR CONTRIBUTIONS

**Xiaoyun Chen:** Writing – original draft (equal). **Yedong Huang:** Writing – original draft (equal). **Fangrong Chen:** Formal analysis (equal). **Hui She:** Writing – review and editing (equal). **Xiangqi Chen:** Writing – review and editing (equal).

## FUNDING INFORMATION

This research was supported by the Science Technology Innovation Joint Project Foundation of Fujian Province (2018Y9038) and Fujian provincial health technology project (2020CXB016).

## CONFLICT OF INTEREST

The authors declare no conflict of interest.

## Data Availability

The data that support the findings of this study are available from the corresponding author upon reasonable request.

## References

[1] Battafarano RJ , Fernandez FG , Ritter J , et al. Large cell neuroendocrine carcinoma: an aggressive form of non‐small cell lung cancer. J Thorac Cardiovasc Surg. 2005;130(1):166‐172. 15999058 10.1016/j.jtcvs.2005.02.064

[2] Lazaro S , Perez‐Crespo M , Lorz C , et al. Differential development of large‐cell neuroendocrine or small‐cell lung carcinoma upon inactivation of 4 tumor suppressor genes. Proc Natl Acad Sci U S A. 2019;116(44):22300‐22306. 31611390 10.1073/pnas.1821745116 PMC6825275

[3] Fasano M , Della CC , Papaccio F , Ciardiello F , Morgillo F . Pulmonary large‐cell neuroendocrine carcinoma: from epidemiology to therapy. J Thorac Oncol. 2015;10(8):1133‐1141. 26039012 10.1097/JTO.0000000000000589 PMC4503246

[4] Travis WD , Brambilla E , Nicholson AG , et al. The 2015 World Health Organization classification of lung tumors: impact of genetic, clinical and radiologic advances since the 2004 classification. J Thorac Oncol. 2015;10(9):1243‐1260. 26291008 10.1097/JTO.0000000000000630

[5] Travis WD , Linnoila RI , Tsokos MG , et al. Neuroendocrine tumors of the lung with proposed criteria for large‐cell neuroendocrine carcinoma. An ultrastructural, immunohistochemical, and flow cytometric study of 35 cases. Am J Surg Pathol. 1991;15(6):529‐553. 1709558 10.1097/00000478-199106000-00003

[6] Eichhorn F , Dienemann H , Muley T , Warth A , Hoffmann H . Predictors of survival after operation among patients with large cell neuroendocrine carcinoma of the lung. Ann Thorac Surg. 2015;99(3):983‐989. 25596870 10.1016/j.athoracsur.2014.10.015

[7] Naidoo J , Santos‐Zabala ML , Iyriboz T , et al. Large cell neuroendocrine carcinoma of the lung: Clinico‐pathologic features, treatment, and outcomes. Clin Lung Cancer. 2016;17(5):e121‐e129. 26898325 10.1016/j.cllc.2016.01.003 PMC5474315

[8] Zhao Y , Castonguay M , Wilke D , et al. Treatment outcomes and incidence of brain metastases in pulmonary large cell neuroendocrine carcinoma. Curr Probl Cancer. 2019;43(1):54‐65. 30107896 10.1016/j.currproblcancer.2018.05.006

[9] Aoyama H , Shirato H , Tago M , et al. Stereotactic radiosurgery plus whole‐brain radiation therapy vs stereotactic radiosurgery alone for treatment of brain metastases: a randomized controlled trial. Jama. 2006;295(21):2483‐2491. 16757720 10.1001/jama.295.21.2483

[10] Wen PY , Loeffler JS : Management of brain metastases. Oncology (Williston Park) 1999, 13(7):941–954, 957–961, 961–962, 9. 10442342

[11] Nayak L , Lee EQ , Wen PY . Epidemiology of brain metastases. Curr Oncol Rep. 2012;14(1):48‐54. 22012633 10.1007/s11912-011-0203-y

[12] Liu CF , Tao YJ . Based on SEER database: population distribution, survival analysis, and prognostic factors of organ metastasis of lung large cell neuroendocrine carcinoma. Front Oncol. 2022;12:810170. 35372078 10.3389/fonc.2022.810170 PMC8971719

[13] Ettinger DS , Wood DE , Aisner DL , et al. NCCN guidelines insights: non‐small cell lung cancer, version 2.2021. J Natl Compr Canc Netw. 2021;19(3):254‐266. 33668021 10.6004/jnccn.2021.0013

[14] Yap WK , Shih MC , Kuo C , et al. Development and validation of a nomogram for assessing survival in patients with metastatic lung cancer referred for radiotherapy for bone metastases. JAMA Netw Open. 2018;1(6):e183242. 30646236 10.1001/jamanetworkopen.2018.3242 PMC6324455

[15] Ceresoli GL , Reni M , Chiesa G , et al. Brain metastases in locally advanced nonsmall cell lung carcinoma after multimodality treatment: risk factors analysis. Cancer‐Am Cancer Soc. 2002;95(3):605‐612. 10.1002/cncr.10687 12209754

[16] Schouten LJ , Rutten J , Huveneers HA , Twijnstra A . Incidence of brain metastases in a cohort of patients with carcinoma of the breast, colon, kidney, and lung and melanoma. Cancer‐Am Cancer Soc. 2002;94(10):2698‐2705. 10.1002/cncr.10541 12173339

[17] Podgrabinska S , Skobe M . Role of lymphatic vasculature in regional and distant metastases. Microvasc Res. 2014;95:46‐52. 25026412 10.1016/j.mvr.2014.07.004 PMC4446725

[18] Roberts N , Kloos B , Cassella M , et al. Inhibition of VEGFR‐3 activation with the antagonistic antibody more potently suppresses lymph node and distant metastases than inactivation of VEGFR‐2. Cancer Res. 2006;66(5):2650‐2657. 16510584 10.1158/0008-5472.CAN-05-1843

[19] Brown M , Assen FP , Leithner A , et al. Lymph node blood vessels provide exit routes for metastatic tumor cell dissemination in mice. Science. 2018;359(6382):1408‐1411. 29567714 10.1126/science.aal3662

[20] Pereira ER , Kedrin D , Seano G , et al. Lymph node metastases can invade local blood vessels, exit the node, and colonize distant organs in mice. Science. 2018;359(6382):1403‐1407. 29567713 10.1126/science.aal3622 PMC6002772

[21] Cao L , Zhao L , Wang M , Zhang XH , Yang ZC , Liu YP . Clinicopathological characteristics and prognosis of pulmonary large cell neuroendocrine carcinoma aged >/=65 years. PEERJ. 2019;7:e6824. 31149394 10.7717/peerj.6824 PMC6532618

[22] Sanchez DCEJ . Diagnosis and treatment of neuroendocrine lung tumors. Arch Bronconeumol. 2014;50(9):392‐396. 24685201 10.1016/j.arbres.2014.02.004

[23] Asamura H , Kameya T , Matsuno Y , et al. Neuroendocrine neoplasms of the lung: a prognostic spectrum. J Clin Oncol. 2006;24(1):70‐76. 16382115 10.1200/JCO.2005.04.1202

[24] Zombori T , Juhasz‐Nagy G , Tiszlavicz L , et al. Large‐cell neuroendocrine carcinoma of the lung ‐ challenges of diagnosis and treatment. Orv Hetil. 2020;161(8):313‐319. 32073294 10.1556/650.2020.31581

[25] Ebben JD , You M . Brain metastasis in lung cancer: building a molecular and systems‐level understanding to improve outcomes. Int J Biochem Cell Biol. 2016;78:288‐296. 27474492 10.1016/j.biocel.2016.07.025 PMC6020150

[26] Shen H , Cao Y , Li X , et al. Surgical intervention improves survival for metastatic non‐small cell lung cancer patients. Medicine (Baltimore). 2016;95(21):e3800. 27227958 10.1097/MD.0000000000003800 PMC4902382

[27] Deng C , Wu SG , Tian Y . Lung large cell neuroendocrine carcinoma: an analysis of patients from the surveillance, epidemiology, and end‐results (SEER) database. Med Sci Monit. 2019;25:3636‐3646. 31095532 10.12659/MSM.914541 PMC6537662

[28] David EA , Canter RJ , Chen Y , Cooke DT , Cress RD . Surgical management of advanced non‐small cell lung cancer is decreasing but is associated with improved survival. Ann Thorac Surg. 2016;102(4):1101‐1109. 27293147 10.1016/j.athoracsur.2016.04.058 PMC5497727

[29] Rossi G , Cavazza A , Marchioni A , et al. Role of chemotherapy and the receptor tyrosine kinases KIT, PDGFRalpha, PDGFRbeta, and met in large‐cell neuroendocrine carcinoma of the lung. J Clin Oncol. 2005;23(34):8774‐8785. 16314638 10.1200/JCO.2005.02.8233

[30] Sun JM , Ahn MJ , Ahn JS , et al. Chemotherapy for pulmonary large cell neuroendocrine carcinoma: similar to that for small cell lung cancer or non‐small cell lung cancer? Lung Cancer. 2012;77(2):365‐370. 22579297 10.1016/j.lungcan.2012.04.009

[31] Ferrara MG , Stefani A , Simbolo M , et al. Large cell neuro‐endocrine carcinoma of the lung: current treatment options and potential future opportunities. Front Oncol. 2021;11:650293. 33937057 10.3389/fonc.2021.650293 PMC8081906

[32] Kawabe T , Yamamoto M , Sato Y , et al. Gamma knife radiosurgery for brain metastases from pulmonary large cell neuroendocrine carcinoma: a Japanese multi‐institutional cooperative study (JLGK1401). J Neurosurg. 2016;125(Suppl 1):11‐17. 27903179 10.3171/2016.7.GKS161459

[33] De Pas TM , Giovannini M , Manzotti M , et al. Large‐cell neuroendocrine carcinoma of the lung harboring EGFR mutation and responding to gefitinib. J Clin Oncol. 2011;29(34):e819‐e822. 22042963 10.1200/JCO.2011.36.2251

[34] Omachi N , Shimizu S , Kawaguchi T , et al. A case of large‐cell neuroendocrine carcinoma harboring an EML4‐ALK rearrangement with resistance to the ALK inhibitor crizotinib. J Thorac Oncol. 2014;9(6):e40‐e42. 24828670 10.1097/JTO.0000000000000103

[35] Hayashi N , Fujita A , Saikai T , et al. Large cell neuroendocrine carcinoma harboring an anaplastic lymphoma kinase (ALK) rearrangement with response to Alectinib. Intern Med. 2018;57(5):713‐716. 29151522 10.2169/internalmedicine.9368-17 PMC5874345

[36] Simbolo M , Mafficini A , Sikora KO , et al. Lung neuroendocrine tumours: deep sequencing of the four World Health Organization histotypes reveals chromatin‐remodelling genes as major players and a prognostic role for TERT, RB1, MEN1 and KMT2D. J Pathol. 2017;241(4):488‐500. 27873319 10.1002/path.4853 PMC5324596

[37] Miyoshi T , Umemura S , Matsumura Y , et al. Genomic profiling of large‐cell neuroendocrine carcinoma of the lung. Clin Cancer Res. 2017;23(3):757‐765. 27507618 10.1158/1078-0432.CCR-16-0355

[38] Fan Y , Ma K , Wang C , et al. Prognostic value of PD‐L1 and PD‐1 expression in pulmonary neuroendocrine tumors. Onco Targets Ther. 2016;9:6075‐6082. 27785054 10.2147/OTT.S115054 PMC5063491

[39] Kim HS , Lee JH , Nam SJ , et al. Association of PD‐L1 expression with tumor‐infiltrating immune cells and mutation burden in high‐grade neuroendocrine carcinoma of the lung. J Thorac Oncol. 2018;13(5):636‐648. 29378266 10.1016/j.jtho.2018.01.008

[40] Chauhan A , Arnold SM , Kolesar J , Thomas HE , Evers M , Anthony L . Immune checkpoint inhibitors in large cell neuroendocrine carcinoma: current status. Oncotarget. 2018;9(18):14738‐14740. 29581877 10.18632/oncotarget.24553 PMC5865703

[41] Zhang X , Sun Y , Miao Y , Xu S . Immune checkpoint inhibitor therapy achieved complete response for drug‐sensitive EGFR/ALK mutation‐negative metastatic pulmonary large‐cell neuroendocrine carcinoma with high tumor mutation burden: a case report. Onco Targets Ther. 2020;13:8245‐8250. 32884302 10.2147/OTT.S259893 PMC7443410

